# Bacterial Factors Targeting the Nucleus: The Growing Family of Nucleomodulins

**DOI:** 10.3390/toxins12040220

**Published:** 2020-03-31

**Authors:** Hélène Bierne, Renaud Pourpre

**Affiliations:** Université Paris-Saclay, INRAE, AgroParisTech, Micalis Institute, Epigenetics and Cellular Microbiology Team, F-78350 Jouy-en-Josas, France; renaudpourpre@gmail.com

**Keywords:** pathogens, effectors, nucleomodulin, nucleus, *Listeria*, epigenetics

## Abstract

Pathogenic bacteria secrete a variety of proteins that manipulate host cell function by targeting components of the plasma membrane, cytosol, or organelles. In the last decade, several studies identified bacterial factors acting within the nucleus on gene expression or other nuclear processes, which has led to the emergence of a new family of effectors called “nucleomodulins”. In human and animal pathogens, *Listeria monocytogenes* for Gram-positive bacteria and *Anaplasma phagocytophilum*, *Ehrlichia chaffeensis*, *Chlamydia trachomatis*, *Legionella pneumophila*, *Shigella flexneri*, and *Escherichia coli* for Gram-negative bacteria, have led to pioneering discoveries. In this review, we present these paradigms and detail various mechanisms and core elements (e.g., DNA, histones, epigenetic regulators, transcription or splicing factors, signaling proteins) targeted by nucleomodulins. We particularly focus on nucleomodulins interacting with epifactors, such as LntA of *Listeria* and ankyrin repeat- or tandem repeat-containing effectors of Rickettsiales, and nucleomodulins from various bacterial species acting as post-translational modification enzymes. The study of bacterial nucleomodulins not only generates important knowledge about the control of host responses by microbes but also creates new tools to decipher the dynamic regulations that occur in the nucleus. This research also has potential applications in the field of biotechnology. Finally, this raises questions about the epigenetic effects of infectious diseases.

## 1. Introduction

Pathogenic or symbiotic bacteria exploit the functions of eukaryotic cells using surface proteins or proteins secreted by general secretion systems or specialized apparatus (Sec, Tat, Type 1 (T1SS) to Type 9 (T9SS) secretion systems, reviewed in [1,2,3]). Interactions between these bacterial factors and various cellular compounds influence multiple signaling events, thus promoting a wide range of effects in host cells, such as cytoskeletal rearrangement, vesicular trafficking, cell proliferation, and survival. It is not surprising that bacteria, like viruses, also employ various strategies to affect the host cell’s gene expression program to their own benefit. For years, most studies have focused on the mechanisms used by bacteria to modify gene expression and cell proliferation through cytosolic signaling pathways. Recently, it has become increasingly clear that bacterial factors can also directly act in the nucleus.

Bacterial targeting of the nucleus was first observed in phytopathogens. In the mid-1970s, Schell and Van Montagu [4] revealed that *Agrobacterium tumefaciens* genetically modifies plants, through integration of its tumor-inducing (Ti) plasmid into the host cell genome. The expression of Ti genes results in uncontrolled proliferation of infected cells and production of nutrients essential to the bacterium’s life. The exploitation of this property led to the first genetically modified organisms of agricultural interest. In the 2000s, Bonas and Lahaye and their teams made another important discovery with the identification of AvrBS3, a protein of *Xanthomonas campestris* acting as a transcription factor in the host cell nucleus [5,6]. AvrBS3 founded the family of transcription activator-like (TAL) effectors [7], which are broadly distributed among *Xanthomonas* and *Ralstonia* species and promote bacterial infection or plant resistance, depending on the host plant species.

While plant pathogens have paved the way to knowledge on nucleus-targeting factors, bacteria responsible for human or animal diseases have not been left out. Within the last ten years, an increasing number of studies highlighted the ability of mammalian bacterial pathogens to also attack the nucleus directly, and thus selectively control the expression of key genes of the immune response. Among the precursory work, research on *Listeria monocytogenes* has shown that a Gram-positive bacterium can secrete a factor that penetrates the nucleus and binds to a protein that regulates the structure of chromatin [8]. In Gram-negative bacteria, the study of Type 3 and Type 4 secretion system (T3SS and T4SS) effectors revealed nucleomodulins with various modes of action, acting either on chromatin or on its regulators. From these data, Bierne and Cossart proposed to group all bacterial factors targeting the nucleus into one family, the nucleomodulins [9]. This term comes from the contraction between “nucleus” and “modulins” (i.e., molecules that modulate the behavior of eukaryotic cells [10]). Since 2012, the number of nucleomodulins has continued to increase, illustrating an incredible sophistication in the mechanisms used by bacteria to act on nuclear factors in the host cell, thus facilitating infection or bacterial colonization [11,12]. In this review, after a brief overview of the complex mechanisms governing the dynamics of gene expression in the nucleus, we recall the data obtained on nucleomodulin paradigms and update the different mechanisms of subversion of nuclear processes, illustrated by a variety of nucleomodulins discovered in both human and animal pathogens. (Please note that the abbreviations of host proteins are listed in Table 1).

## 2. Nuclear Processes

Most animal and plant cells have a nucleus containing their genome. This compartment, which is delimited by a double-membrane envelope, has given them their name—eukaryotic cells (from the Greek εὖ, “well/true”, and κάρυον, “nut/kernel”). Within the nucleus, the DNA is condensed into a nucleoprotein complex called chromatin. The first level of organization is the nucleosome, where 147 base pairs of DNA are wrapped twice around an octamer of histones (i.e., the (H3–H4)2 tetramer and two H2A–H2B dimers). The nucleosomes and their associated proteins form a fiber, the compaction of which plays a major role in gene expression. Decondensed euchromatin is permissive to transcription, in contrast to compacted heterochromatin. At the genomic level, chromatin is intricately organized through the establishment of tertiary structures and loops. This complex three-dimensional (3D) organization is crucial to the establishment of the transcriptional programs necessary for cell differentiation and embryonic development but also for the response of somatic cells to external stimuli [13]. 

Gene expression not only depends on DNA-binding transcription factors but also on post-translational modifications (PTMs) of chromatin, a mechanism of control often referred to as “epigenetic regulation”. Chromatin modifications include DNA methylation [14] and histone PTMs (e.g., acetylation, methylation, phosphorylation, ubiquitination, sumoylation, cADP-ribosylation, crotonylation) [15,16]. Specific combinatorial modifications control the chemical properties of nucleosomes and the recruitment of chromatin regulators, organizing them into large multiprotein chromatin-remodeling complexes. The modular, multifunctional, and combinatorial nature of these complexes ensures extremely precise temporal and spatial control over chromatin structure. When inherited, chromatin marks allow somatic cells to retain their specificity as tissues are renewed. However, these marks can also be more labile and change gene expression transiently, under the effect of endogenous or exogenous stimuli. For simplicity, the molecular components of the chromatin-remodeling machinery will be referred to here as “epifactors” and chromatin modifications as “epigenetic marks”.

In addition to transcription, the splicing of precursor messenger RNAs (pre-mRNAs) by the spliceosome [17], as well as processes requiring access to DNA, such as DNA replication, recombination, and repair, are intimately linked to chromatin configuration. Their respective cellular machineries work in close association with chromatin-remodeling complexes.

## 3. Nucleomodulins of *Listeria monocytogenes*

The foodborne pathogen *L. monocytogenes* is the agent of listeriosis, a severe disease that particularly affects immunocompromised individuals and the fetuses of pregnant women [18]. This Gram-positive bacterium invades many cell types and affects various organs such as the liver, spleen, placenta, and brain. Studies on *Listeria* have been particularly useful in identifying various mechanisms of host–pathogen interaction [19,20], in particular, chromatin changes induced by bacteria [12,21]. One of these mechanisms involves a virulence protein secreted by the general Sec secretion system, *Listeria* nuclear targeted protein A (LntA), the first protein of this pathogen detected in the nucleus [8]. LntA is a small basic protein organized into five alpha helixes and presenting a putative nuclear localization signal (NLS) in its central part [22]. Its human binding partner is BAHD1, named as such due to the presence of a C-terminal BAH domain. The BAH domain is found in several proteins that interact with chromatin [23]. As BAHD1 was of an unknown function at the time of its discovery as an LntA target [24], the first step was to characterize it. Several studies showed that BAHD1 acts by compacting chromatin into heterochromatin, leading to transcriptional repression. It exerts its function in a chromatin-remodeling complex comprising heterochromatin proteins (HP1), histone methyltransferases (e.g., G9a), and histone deacetylases (e.g., HDAC1/2) [8,24,25].

**Figure 1:**
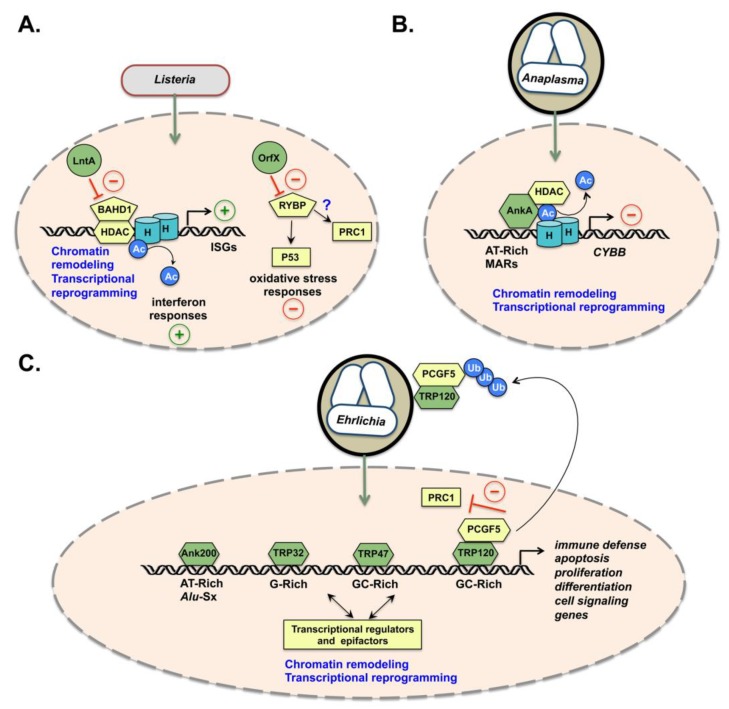
Nucleomodulins of *L. monocytogenes*, *A. phagocytophilum*, and *E. chaffeensis*. *Listeria* in the cytosol and *Anaplasma* or *Ehrlichia* within vacuoles secrete nucleomodulins (in green) that enter into the nucleus (represented by an orange oval; blue cylinders labeled “H” represent histones; host nuclear factors are in yellow). Post-translational modifications (PTMs): acetylation (Ac), ubiquitination (Ub). (**A**) *L. monocytogenes* secretes two nucleomodulins. LntA inhibits the binding of a BAHD1–HDAC chromatin repressive complex to ISG promoters, thus promoting histone acetylation and activation of ISG expression and stimulating interferon responses. OrfX decreases RYBP amounts, which impairs P53-mediated activation of the oxidative stress response and potentially PRC1-mediated regulation. (**B**) *A. phagocytophilum* AnkA binds to AT-rich DNA motifs and recruits HDAC1 at the *CYBB* promoter, thus promoting histone deacetylation and repression of *CYBB* and altering the function of NAPDH oxidase. AnkA also binds to nuclear matrix attachment regions (MARs) throughout the genome. (**C**) *E. chaffeensis* secretes four nucleomodulins. Ank200 binds to *Alu-Sx* DNA motifs, while TRP32, TRP47, and TRP120 bind to G-rich or G + C-rich DNA motifs, as well as to a set of transcriptional regulators and epifactors, leading to deregulation of many host genes with multiple functions. In particular, TRP120 interacts with PCGF5 of the PRC1 complex. Delocalization and ubiquitination of PCGF5 by TRP120 at the ehrlichial vacuole is proposed to inhibit PRC1-mediated repression of a set of host genes.

The genes inhibited by BAHD1 differ depending on the cells and the signals to which they are exposed. In the case of *Listeria* infection of epithelial cells, BAHD1 represses the expression of genes stimulated by interferons (ISGs), particularly interferon lambda [8]. When bacteria secrete LntA, this nucleomodulin enters the nucleus, where it inhibits the recruitment of BAHD1 and HDACs to ISG promoters, thus triggering histone acetylation and activation of ISGs (Figure 1A). The three-dimensional structure of LntA and directed mutagenesis identified a dilysin motif (K180/K181) of LntA as crucial for the LntA–BAHD1 interaction [22]. Mutation of K180/K181 in LntA abolishes this interaction and the function of LntA as an activator of the interferon response during infection. Deletion or overexpression of the *lntA* gene, as well as haplo-deficiency in *Bahd1*, impedes *L. monocytogenes* infection in mice [8]. This suggests that the finely regulated production of LntA by the bacteria and, thus, the controlled manipulation of BAHD1 during infection, modulates the interferon response to control bacterial colonization in the host [26]. However, the signals that trigger the expression of *lntA* by bacteria in vivo remain unknown. Interestingly, the knock-out of the *Bahd1* gene causes a defect in placental growth in mice, indicating a key role of BAHD1 in the development of this organ [25]. This observation opens the possibility that manipulation of the BAHD1 epifactor by *Listeria* may contribute to maternal–fetal listeriosis.

Recently, OrfX was a second nucleomodulin identified among the virulence proteins secreted by *L. monocytogenes* [27]. OrfX interacts with RYBP, a multifunctional zinc finger nuclear protein that interacts with several transcription factors and epifactors [28]. RYBP also binds to the E3 ubiquitin ligase MDM2 and inhibits MDM2-mediated degradation of the transcription factor P53 [29]. P53 has multiple cellular roles, including the regulation of reactive oxygen (ROS) and reactive nitrogen species (RNS) levels. In the context of *Listeria* infection of macrophages, OrfX binds to and decreases the level of RYBP, which contributes to bacterial survival by altering the production of superoxide and nitric oxide. It has been proposed that OrfX indirectly modulates ROS production by altering the P53–MDM2 pathway via its interaction with RYBP [27] (Figure 1A). This model remains to be clarified, as well as the potential effect of OrfX on RYBP-associated epifactors, such as the polycomb repressive complex 1 (PRC1) [28].

In conclusion, the study of *Listeria* nucleomodulins identified two main types of mechanisms in the nucleus: the targeting of chromatin or components of chromatin-remodeling complexes and the control of stability/degradation of other nuclear regulatory proteins.

## 4. Ankyrin Repeat- and Tandem Repeat-Containing Nucleomodulins: The *Anaplasma phagocytophilum* and *Ehrlichia chaffeensis* Paradigms

Protein domains containing repeats are often involved in protein–protein interactions. Among these, ankyrin repeats (Ank) are the most common protein–protein interaction motifs in nature [30]. Several Gram-negative intracellular pathogens, such as *Anaplasma*, *Ehrlichia*, *Rickettsia*, *Orientia*, *Coxiella*, and *Legionella* encode Ank-containing proteins, among which are nucleomodulins with various roles. The virulence factor Ankyrin A (AnkA) of *A. phagocytophilum* is a paradigm of this family. *A. phagocytophilum* is an obligate intracellular rickettsial pathogen that causes human granulocytic anaplasmosis [31]. This bacterium infects neutrophils, where it replicates within vacuoles and secretes effectors by a T4SS. The AnkA effector localizes in the cytosol and the nucleus, where it binds AT-rich DNA regions and modulates the transcription of antimicrobial defense genes, such as *CYBB*, encoding the subunit beta (NOX2) of the NADPH oxidase [32]. AnkA acts in an opposite manner to LntA, by recruiting rather than inhibiting an HDAC-associated complex. The mechanism of action involves AnkA-mediated recruitment of HDAC1 to the *CYBB* promoter, leading to the deacetylation of histone H3 and repression of *CYBB* expression [33,34,35] (Figure 1B). The production of superoxide anion by the NADPH oxidase is then hindered and deprives infected neutrophils of a key process in the elimination of bacteria. 

Interestingly, AnkA targets AT-rich sequences distributed on distinct chromosomes and exhibits characteristics of a protein that binds to the attachment regions of the nuclear matrix (MAR-binding). Thus, AnkA functionally mimics the MAR-binding protein SATB1, which also binds to the *CYBB* promoter and represses its transcription by recruiting HDACs [35,36]. ChIP-seq experiments have reported a distribution of AnkA in the vicinity of promoters, intergenic regions and lamina-associated MARs [37]. Regarding the role of MAR-binding proteins in nuclear matrix attachment, spatial organization of chromatin and large-scale transcriptional regulation [38], AnkA could act as a “reprogrammer” of the neutrophil genome. This introduces the hypothesis that bacterial effectors such as AnkA could act as global genome organizers both acting in *cis* (locally) and *trans* (at a distance) to a target gene. By controlling the dynamics of chromosomal looping, they may change the 3D structure of chromatin. Like AnkA, other nucleomodulins could act on chromatin anchoring factors. For example, SinC, secreted by *Chlamydia psittaci*, targets proteins of the inner membrane of the nucleus, such as MAN1 and LAP1. SinC could act on their function as chromatin organizers via interaction with lamins [39].

Another important paradigm in the field of nucleomodulin research is *E. chaffeensis*, the causative agent of human monocytotropic ehrlichiosis [40]. Like *Anaplasma, E. chaffeensis* is an obligate intracellular bacterium belonging to the order of Rickettsiales. *E. chaffeensis* reprograms the mononuclear phagocyte using several effectors, including a set of T1SS nucleomodulins [41]: an Ank repeat-containing protein, Ank200, and several serine-rich tandem repeats-containing proteins (Trp) (Figure 1C). Ank200 interacts with chromatin at repetitive AT-rich DNA regions, called *Alu*-Sx elements, within the promoters and the intergenic region of genes involved in a wide range of functions (e.g., transcriptional and translational regulation, immune response and cell signaling, intracellular trafficking and cytoskeletal rearrangement). The majority of Ank200 target genes are downregulated during infection, although a few, such as those encoding TNF-α and the interferon response pathway activator STAT1, are upregulated [42]. These observations support the hypothesis of large-scale transcriptional alteration induced by Ank200 via mechanisms associated with the regulation of *Alu*-Sx elements. TRP32 and TRP120 bind to host-cell DNA at G-rich and G + C-rich motifs, respectively, as well as chromatin-associated proteins, such as histone methylases and demethylases, polycomb-group (PcG) proteins and other components of chromatin-remodeling complexes [43,44,45,46,47,48]. TRP120 acts as a transcriptional activator for host genes [44], while TRP32 activates or represses expression of its target genes [46,47]. Recently, another TRP effector, TRP47, has been identified as a fourth *E. chaffeensis* nucleomodulin [48]. TRP47 translocates into the nucleus via a MYND (myeloid, Nervy, and DEAF-1)-binding domain and targets multiple infection-associated genes by binding DNA at G + C-rich motifs. It can be noted that the MYND domain, which is a zinc finger motif that primarily functions as a protein–protein interaction module, is present in several host chromatin-remodeling proteins [48,49].

A functional analysis of the genes targeted by the different TRPs revealed specificities, but also redundancies, in the regulation of genes and cellular pathways, essentially involved in immune response, apoptosis, cell differentiation, and proliferation. Overlap in the function of target genes could be a strategy developed by *E. chaffeensis* to secure the modulation of certain cellular signaling during infection. The mechanism by which ehrlichial nucleomodulins bind to DNA involves disordered tandem repeats that fold into an ordered structure when bound to DNA [50]. In addition to its binding to DNA, TRP120 interacts with several epifactors, including the RING domain of PCGF5 [51], a component of a PRC1-like complex. Furthermore, a HECT E3 (Homologous to E6AP C-terminal ubiquitin ligase) domain is present in the C-terminal region of TRP120 and targets PCGF5 for poly-ubiquitination [52]. Recent results support a model in which, during *E. chaffeensis* infection, TRP120 relocalizes PCGF isoforms from the nucleus to the ehrlichial vacuole and induces their degradation [53] (Figure 1C). This coincides with a decrease in the level of the PRC1-mediated epigenetic mark H2AK119Ub (histone H2A ubiquitinated at lysine 119) and in the transcriptional activation of PRC1 target genes, such as homeobox protein (HOX) encoding genes [53]. Knockdown of PCGF5 by small interfering RNAs (siRNAs) results in a significant increase in *E. chaffeensis* infection. These studies highlight an original strategy of *E. chaffeensis* to manipulate epifactors by interactions between TRP120 and PCGF5 isoforms to promote infection.

The example of TRP120 illustrates the multifunctionality of certain nucleomodulins in targeting DNA (by protein–DNA interactions) and/or nuclear factors (by protein–protein interactions) together with modifying targets via enzymatic domains.

## 5. Nucleomodulins Acting as Chromatin-Modification Enzymes

PTMs play a key functional role in the execution of many cellular processes, such as the regulation of subcellular localization, conformation, stability, enzyme activity, or protein interactions, as well as signal transduction and regulation of gene expression. Various pathogens have tamed these modifications and their regulations to interfere with cellular functions and promote infection [54]. Some nucleomodulins are modifying enzymes, which functionally mimic eukaryotic enzymes or innovate by creating specific modifications. A first example comes from the study of protein nuclear effector (NUE), a T3SS effector of the obligate intracellular human pathogen *Chlamydia trachomatis*. NUE is the first bacterial factor described as a mimic of host histone methyltransferases. Indeed, NUE contains a SET domain commonly found in many eukaryotic methyltransferases [55] and acts as a lysine methyltransferase for host histones H2B, H3, and H4 [56,57]. NUE was found in the nuclear fraction of HeLa cells infected with *C. trachomatis* and was shown to associate with chromatin, but its target genes remain unknown [57]. 

**Figure 2:**
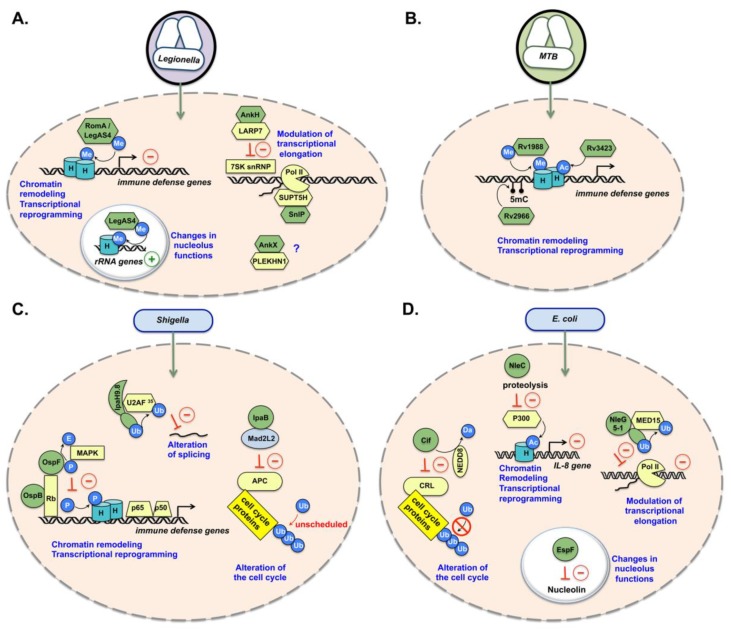
Nucleomodulins of *L. pneumophila*, *M. tuberculosis (Mtb)*, *S. flexneri*, and *E. coli*. *Legionella* or *Mtb* within vacuoles and *S. flexneri* or *E. coli* from the extracellular compartment (or the entry vacuole, for *Shigella*) secrete nucleomodulins (in green) that enter into the nucleus (represented by an orange oval; blue cylinders labeled “H” represent histones; host nuclear factors are in yellow). PTMs: acetylation (Ac), methylation (Me), phosphorylation (P), eliminylation (E), ubiquitination (Ub), deamination (Da). (**A**) *L. pneumophila* secretes four nucleomodulins. The histone methyltransferase RomA/LegAS4 triggers H3K14me3 at specific gene promoters, and thus, transcriptional repression of a network of immune defense genes. An additional function was described for LegAS4 in the nucleolus, where it binds to HP1 at rDNA promoters and activates rDNA gene expression. AnkH interacts with LARP7, a component of the 7SK snRNP complex, and interferes with transcriptional elongation by RNA Pol II. SnlP interferes with RNA Pol II by inhibiting the transcription elongation factor SUPT5H. AnkX interacts with PLEKHN1 but the function of this interaction is unknown. (**B**) *M. tuberculosis* (MTB) secretes three nucleomodulins playing a role in the deregulation of immune defense gene expression. Rv1988 is a histone methyltransferase that triggers H3R42me2. Rv3423 is a histone acetyltransferase. Rv2966c is a DNA methyltransferase. (**C**) *S. flexneri* secretes four nucleomodulins. OspF induces elimination of mitogen activated protein (MAP)-kinases in the nucleus, thus preventing phosphorylation of histone H3 on serine 10 and inducing repression of a subset of immune defense genes. OspF also binds the retinoblastoma protein (Rb), as another effector, OspB. IpaH9.8 ubiquitinylates and promotes degradation of the splicing factor U2AF^35^. IpaB has pleiotropic functions, one of which involves Mad2L2, an inhibitor of the APC^cdh1^/C ubiquitin ligase complex. IpaB interaction with Mad2L2 promotes unscheduled activation of APC and premature degradation of several cell cycle proteins. (**D**) *E. coli* secretes four nucleomodulins. NleG5-1 ubiquitinylates and promotes degradation of the mediator complex component MED15. Cif deamidates NEDD8 in the CULLIN subunit of SCF ubiquitin ligase complexes, thus preventing SCF-mediated degradation of cell cycle regulators. NleC is a metalloproteinase targeting histone acetyltransferase (HAT) p300 for degradation. EspF targets the nucleolus and causes loss of nucleolin.

Following this pioneering discovery, a T4SS effector with a SET domain and Ank repeats was identified in *Legionella pneumophila*. This nucleomodulin was named, depending on the producing strain, RomA (in strain Paris) [58] or LegAS4 (in strain Philadelphia) [59]. RomA/LegAS4 is a histone methyltransferase with different activities and substrates depending on its intranuclear location. In the nucleoplasm, RomA and LegAS4 (which show 100% identity in their SET domain) tri-methylate histone H3 on lysine 14, a modification that does not exist physiologically in mammals [58,60] (Figure 2A). 

The increase in H3K14me3 decreases the level of acetylation of the same residue, leading to transcriptional repression. Immune response genes are affected, allowing the efficient replication of bacteria in macrophages [58]. Furthermore, for LegAS4, it was shown that this protein can also activate the expression of ribosomal RNAs, when localized in the nucleolus [59] (Figure 2A). In particular, LegAS4 targets rDNA promoters and intergenic regions via its interaction with HP1α/γ. Interestingly, *Burkholderia thailandensis* produces a LegAS4-like protein, BtSET, which performs the same function [59], suggesting that rDNA targeting could be a common strategy for several pathogenic bacteria. Importantly, both activities of RomA/LegAS4 contribute to *Legionella* infection efficiency in macrophages [58,59]. Overall, these results highlight that bacterial SET-domain proteins are one of the bacterial tools used by pathogens to induce epigenetic changes in the host to favor infection.

The pathogenic actinomycetes responsible for tuberculosis, *Mycobacterium tuberculosis* (*Mtb*) evolved diverse strategies to directly manipulate chromatin in the nucleus by producing distinct modifiers (Figure 2B). Rv1988 acts as a histone methyltransferase in a unique way, by di-methylating a non-canonical arginine residue (R42) in the core region of histone H3 (H3R42) [61]. The H3R42me2 modification induces the repression of genes involved in oxidative stress, such as *NOX1* and *NOX4*, which correlates with decreased ROS activity in macrophages during infection. The nucleomodulin Rv3423 is a histone acetyltransferase (HAT), acetylating H3 on lysine 9 (H3K9) or lysine 14 (H3K14) [62]. *Mtb* also secretes a DNA methyltransferase, Rv2966c, acting in a non-CpG context and targeting specific genes via its interaction with epigenetic markers [63]. To date, no other bacterial DNA methyltransferases (DNMTs) targeting host DNA have been described, except in *Mycoplasma hyorhinis*, an intracellular commensal bacterium that may act as an opportunistic pathogen. *M. hyorhinis* possesses three DNA methyltransferases: Mhy1 and Mhy2, catalyzing CG methylation, and Mhy3, which acts at GATC sites [64]. These DNMTs can selectively methylate the host genome at sites free of pre-existing endogenous methylation, including those of various cancer-associated genes. The role of this specific GATC methylation is not known but could constitute an epigenetic signature of an infection.

## 6. Nucleomodulins Triggering PTM on Nuclear Regulators

Pathogenic bacteria have also evolved mechanisms to modify nuclear components other than histones or DNA. Several T3SS effectors of bacterial enteropathogens *S. flexneri* and *E. coli* accurately illustrate these mechanisms. *S. flexneri* OspF is a phosphothreonine lyase that permanently modifies host mitogen-activated protein kinases (MAPKs) by a PTM termed “elimination” [65,66]. This reaction converts a phosphothreonine residue into a dehydrobutyrin residue, which cannot be phosphorylated afterward. OspF locks MAPKs in the nucleus in an inactive state, thus inhibiting histone H3 phosphorylation on serine 10 at promoters regulated by NF-κB, and subsequently, the expression of pro-inflammatory genes (Figure 2C). When compared to a wild-type strain, an *ospF*-deficient mutant stimulates inflammation and epithelial destruction in the rabbit ileal loop model [66]. OspF also interacts with the chromatin reader HP1γ and hinders the localization of HP1γ to chromatin, as a result of HP1γ dephosphorylation at serine 83 [67]. This delocalization is associated with a fine deregulation of HP1γ target genes. Furthermore, OspF and another nuclear-targeted effector, OspB, interact with the human retinoblastoma protein, which is known to bind several chromatin-remodeling factors [68] (Figure 2C). *Shigella* likely uses OspF–OspB synergy to downregulate host innate immunity via alteration of the chromatin structure at specific genes. 

*S. flexneri* also secretes a family of T3SS effectors with E3-ubiquitin ligase activity, called IpaHs [69,70]. The study of IpaH9.8 built a novel family of bacterial E3-ligases targeting several host cytosolic and nuclear proteins for proteasome-dependent degradation [71]. In the nucleus, IpaH9.8 targets a splicing factor, U2AF35 (or U2AF1), suggesting that it may interfere with mRNA splicing [72,73,74] (Figure 2C). IpaH enzymes are a family of bacterial effectors characterized by a particular N-terminal leucine-rich repeat (LRR) domain, referred to as the LPX domain [75], which is involved in substrate recognition, and a C-terminal E3 ubiquitin ligase domain, named NEL (novel E3 ligase) [75,76,77]. The NEL domain has an original structure that differs from known eukaryotic models but retains the catalytic cysteine characteristic of E3, demonstrating functional mimicry. It is found in other bacterial proteins, such as *Salmonella enterica* nucleomodulin SspH1, which induces the degradation of PKN1 kinase and the inhibition of NF-κB-dependent pro-inflammatory genes [71,78]. Ssph1 also acts on the signaling pathway dependent on androgen receptors that regulate the function and activation of neutrophils and macrophages [79].

Pathogenic *Yersinia* species *Y. pestis*, *Y. pseudotuberculosis* and *Y. enterocolitica* secrete a nucleomodulin related to IpaH and Ssph1 [75]. *Yersinia* outer protein M (YopM) possesses a LPX-type domain and traffic to the nucleus [80,81]. However, although it has been proposed to function as an E3 ubiquitin ligase for one particular strain of *Y. pestis* [82], YopM does not possess an NEL domain and appears to rather act as a scaffolding protein facilitating the formation of a complex between its targets, the serine/threonine kinases RSK1 and PKN2 [83,84]. YopM causes enhanced phosphorylation of RSK1 in the nucleus, leading to enhanced transcription of immunosuppressive cytokine genes, such as *IL-10* [85]. YopM intranuclear levels are dependent on its interaction with RNA helicase DDX3 and this nucleocytoplasmic shuttle determines the level of RSK1 phosphorylation and *IL-10* transcription [86]. 

Pathogenic *E. coli* also produce nucleomodulins that act on ubiquitination and degradation of target nuclear proteins. NleG5-1, a T3SS effector of enterohaemorrhagic (EHEC) *E. coli*, contains an ubiquitin ligase U-box domain. In the nucleus, NleG5-1 induces the degradation of a member of the Mediator complex, MED15, which is a master coordinator of RNA polymerase II (Pol II)-dependent transcription [87] (Figure 2D). Recently, *Orientia tsutsugamushi*, the causative agent of bush typhus, was shown to produce a family of Ank proteins (1U5, 1A, 1B, 1E, 1F, 1U4, 1U9) also engaged with the host ubiquitination machinery. These effectors, which contain N-terminal Ank repeats and a C-terminal F-box-like domain, also known as the PRANC (pox protein repeats of ankyrin C terminus) motif [88], localize to the nucleus upon ectopic expression in human cells [89]. They interact with CULLIN-1 and SKP1, two members of a multiprotein E3 ubiquitin ligase complex. It is proposed that the Ank domain binds to a host cell-specific target substrate, while the F-box recruits SKP1 to nucleate the ubiquitin ligase complex that promotes substrate degradation. One of these potential nucleomodulins, Ank1U5, appears to degrade the transcription factor EF1α [89]. However, the impact on target genes is still unknown.

Pathogenic *E. coli* strains secrete cell cycle-modulatory factors called cyclomodulins [90]. One of these, Cif, is a deamidase that targets the ubiquitin-like protein NEDD8 [91,92,93,94]. Cif-mediated deamidation of NEDD8 abolishes the activity of CULLIN-RING ubiquitin ligases (CRLs) on multiple substrates, including key cell cycle regulators (Figure 2D). Consequently, Cif induces cell-cycle arrest in enteropathogenic *E. coli* (EPEC)-infected cells [93]. It can be noted that proteins homologous to Cif proteins have been found in several other Gram-negative pathogenic species (*Yersinia pseudotuberculosis*, *Photorhabdus luminescens*, *Photorhabdus asymbiotica*, and *Burkholderia pseudomallei*) [95]. Interestingly, this mode of action converges with that of the *S. flexneri* effector IpaB [96]. IpaB has pleiotropic functions in cells infected with *S. flexneri*, including the mediation of cell-cycle arrest upon binding to MAD2B, an inhibitor of the APC/C^cdh1^ ubiquitin ligase complex. IpaB localizes in the nucleus during the G2/M phase, when overexpressed in Hela cells, and promotes unscheduled activation of APC/C^cdh1^ and premature degradation of APC/C^cdh1^ substrates, resulting in a delay in mitotic progression (Figure 2D). Intestinal crypts infected with an *ipaB* mutant have a higher number of progenitor cells and are less colonized than with the wild-type *Shigella* strain. These findings suggest that one of the various functions of IpaB is to slow down epithelial cell turnover and exfoliation, enabling *Shigella* to persist in the epithelial mucosa [96].

## 7. Nucleomodulins Acting as Proteases

Another strategy involves direct proteolytic cleavage. EHEC NleC is a metalloprotease capable of reducing the amount of histone acetyltransferase p300 in the nucleus, thereby attenuating the expression of the pro-inflammatory cytokine *IL-8* gene [97] (Figure 2D). *Salmonella *enterica** serovar Typhimurium also secretes metalloproteases: PipA, GogA, and GtgA [98,99]. These enzymes target the nucleus and degrade subunits of NF-κB p65, RelB, and cRel. The deletion of their encoding genes induces overactivation of the NF-κB response during *Salmonella* infections, demonstrating a control of the inflammatory response. In *Neisseria meningitidis*, a major cause of bacterial meningitis and septicemia, the adhesion and penetration protein (App) and meningococcal serine protease A (MspA) are type Va autotransporters harboring S6-family serine endopeptidase domains. App and MspA are internalized by human cells and traffic to the nucleus. In vitro, MspA and App proteolytically cleave histone H3, and in vivo, they induce a dose-dependent increase in dendritic cell death via caspase-dependent apoptosis [100]. However, the functional link between these properties remains to be clarified.

## 8. Ever More Substrates and Functions: An Expanding Family

Over the years, the number of nucleomodulins has grown steadily, demonstrating a variety of mechanisms and substrates. In particular, more bacterial Ank proteins have been shown to play a role in the nucleus. Recently, two other *O. tsutsugamushi* Ank nucleomodulins were described to act on another key nuclear process: protein shuttling through the nuclear envelope. Ank1 and Ank6 are transported into the nucleus via the classical importin-dependent pathway β1, and then induce export of the p65 subunit of NF-κB via exportin 1. Thus, Ank1 and Ank6 inhibit the accumulation of p65 in the nucleus and the transcription of pro-inflammatory genes of the NF-κB, pathway [101]. 

In *L. pneumophila*, the AnkH effector has recently been shown to interact with the host nuclear protein LARP7, which is a component of the 7SK small nuclear ribonucleoprotein (snRNP) complex. The AnkH–LARP7 interaction interferes with transcriptional elongation by RNA Pol II, leading to the global reprogramming of transcription of infected macrophages and contributing to the proliferation of *Legionella* [102] (Figure 2A). *Legionella* nucleomodulin SnlP illustrates another way to control RNA Pol II. SnlP targets and inhibits SUPT5H, a component of the DRB sensitivity-inducing factor (DSIF) complex that regulates mRNA processing and transcription elongation by RNA Pol II [103] (Figure 2A). Ectopic expression of SnpL leads to massive upregulation of host gene expression and macrophage cell death. *L. pneumophila* also secretes AnkX, which may have other functions related to the manipulation of nuclear factors. Under ectopically expressed conditions, mCherry-AnkX exhibits a punctuated distribution in the nucleus and co-localizes with PLEKHN1, a poorly characterized protein that localizes in nuclear speckles and cytosolic puncta [104] (Figure 2A).

Studies on *Coxiella burnetii*, the zoonotic agent of Q fever, have brought to light a T4SS Ank effector involved in the control of apoptosis: AnkG. The import of AnkG into the nucleus is induced by the stress of infection and requires the interaction of AnkG with p32/C1qBP and importin α1. The antiapoptotic function of AnkG is strictly dependent on its nuclear location [105]. One model speculates that AnkG first targets p32/C1qBP at the mitochondrion in order to “sense” the apoptotic stress and then migrate and perform its function within the nucleus in order to block stress-induced apoptosis [106]. However, the nuclear targets and mechanism of action of AnkG remain unknown. The Gram-positive bacterium *Streptococcus pyogenes* expresses a serine/threonine phosphatase, SP-STP, which also plays a role in apoptosis by targeting the nucleus [107]. It has been proposed that SP-STP influences transcription of apoptotic genes in pharyngeal cells.

It is interesting to note that within the nucleus, the nucleolus can also be controlled by pathogens [108], as shown above with *Legionella* LegAS4 (Figure 2A) [59]. The *E. coli* effector EspF also targets the nucleolus, where it disrupts a subset of nucleolar factors. In particular, driven by an N-terminal nucleolar-targeting domain, EspF causes the complete loss of nucleolin, the most abundant nucleolar protein (Figure 2D) [109].

## 9. Regulation of Nucleomodulins by PTMs

Many bacterial proteins are subject to chemical modifications by the eukaryotic post-translational modification machinery. This contributes to their function and promotes infection, or conversely, inactivates them as part of the host cell’s defense mechanisms. Nucleomodulins are no exception. We briefly cite a few examples. *Chlamydia* NUE displays automethylation activity, which improves its ability to methylate histone substrates [57]. *Anaplasma* AnkA is phosphorylated on tyrosine by human Abl-1 kinase, the expression of which is stimulated during infection. The results suggest that Abl-1 is necessary for optimal infection [110]. *Ehrlichia* TRP32 is regulated by different modifications. Tyrosine phosphorylation controls its nuclear localization [47], while ubiquitination on lysine 63, catalyzed by the host cell protein NEDD4L, is required for TRP32 transcription factor function and subnuclear localization [111]. For TRP120, ubiquitination and sumoylation are critical in establishing its eukaryotic target landscape and thus, function [51,52]. In *Mtb*, phosphorylation of nucleomodulin Rv2966c increases its ability to interact with DNA and stimulates its methyltransferase activity [63]. The nuclear localization of *Shigella* nucleomodulin OspF requires sumoylation at lysine 19. Mutation of this sumoylation site abolishes OspF localization to the nucleus and OspF-mediated dephosphorylation of histone H3 [112].

## 10. Conclusions

Nucleomodulins are sophisticated weapons in the toolbox of bacterial pathogens, as summarized in Table 2. It is remarkable that some of them have evolved as mimics of eukaryotic nuclear proteins, either through sequence homologies (e.g., ANK, LRR, MYND, SET, F-box, U-box) or through true structural innovations (e.g., NEL domain). Interestingly, computational biology approaches suggest the presence of many uncharacterized proteins with potential nucleomodulin domains in the genomes of intracellular bacteria, such as *Anaplasma* [113], *Legionella* [114], and other species [115,116]. This opens up an important field of research into their functional roles. It should be noted that nucleomodulins, like many toxins and effectors, often have several different ligands and subcellular localizations. Thus, they can perform multiple functions, as shown for instance for effectors with ubiquitin ligase or methyltransferase activities [70,75,117]. Nucleomodulin diversity is also gradually being uncovered in plant pathogenic bacteria, as well as in protozoa and fungi. They also demonstrate elegant strategies for subverting nuclear processes, sometimes with functional convergence with bacterial pathogens. For example, *L. monocytogenes* LntA [8] and *Toxoplasma gondii* Tglst [118] show significant convergence in modulating interferon responses by targeting functionally related chromatin-remodeling complexes, BAHD1 and NURD (nucleosome remodeling and deacetylase complex), respectively. 

Nucleomodulins are also promising tools for understanding the biology of the nucleus. For example, the study of *Listeria* nucleomodulin LntA has led to the characterization of BAHD1 as a new epigenetic regulator [21]. The study of nucleomodulin target genes could also lead to the discovery of new pathways involved in infectious diseases and potential targets for pharmacological intervention. Similarly to the exploitation of *Xanthomonas* TAL effectors [7] and *Agrobacterium* Ti plasmid, some nucleomodulins could be used in biotechnology. For example, the engineering of bifunctional proteins, such as IpaH proteins, has led to the creation of tools called ubiquibodies, which are composed of a synthetic binding protein fused to a ubiquitin ligase E3, allowing ubiquitination and post-translational degradation of a target protein. IpaH9.8-based ubiquibodies are a novel proteome editing technology with the potential to pharmacologically modulate disease-causing proteins [119]. 

The characterization of nucleomodulins has considerably increased over the past ten years, but many questions remain. How does a pathogen organize the secretion and function of several nucleomodulins in time and space during infection? With at least four nucleomodulins, *E. chaffeensis*, *L. pneumophila*, *S. flexneri,* and *E. coli* illustrate this complexity. It is tempting to speculate that a multiplicity of modifications of nuclear processes carried out by the same bacterium is an effective strategy for maintaining a replication or persistence niche in the host. However, there is still a lack of data on the expression of these nucleomodulins during the different phases of the disease and in clinical or environmental strains. In addition, the results have so far been most often obtained in cultured cells. It is now necessary to develop more investigations in vivo. However, the absence of an appropriate animal model can sometimes be limiting. Furthermore, it remains difficult to genetically manipulate obligate intracellular bacteria, which makes it difficult to search for altered phenotypes.

Another important question concerns the duration of the phenomena induced by nucleomodulins in the nucleus. For effectors generating epigenetic modifications, we do not yet have precise information on the maintenance of these modifications in the host cell and its progenies, and on the consequences on long-term reprogramming of host gene expression. In order to determine this impact, studies should be carried out at both the tissue and cellular level to identify the fate of infected cells. For bacteria targeting innate immune cells, such as macrophages (e.g., *Legionella, Ehrlichia, Mtb*) and neutrophils (e.g., *Anaplasma*), one can ask the question of the impact of nucleomodulins on the innate immune memory. Indeed, it has recently been shown that certain innate immune cells can remember previous encounters with pathogens, through maintenance of specific epigenetic marks. Do nucleomodulins contribute to this phenomenon, termed “trained immunity” [120]? All these issues indicate that much remains to be done to unravel all the mysteries of the interactions between bacteria and the nucleus, which opens the way to exciting new research.

## Figures and Tables

**Table 1:** List of host protein abbreviations.

Name	Full Name
APC/C^cdh1^	Anaphase-promoting complex/cyclosome adaptor protein CDH1
BAHD1	Bromo adjacent homology domain-containing 1
CYBB	Cytochrome b beta
DDX3	DEAD-box helicase 3 X-linked
EF1α	Transcription factor E1F alpha
G9a	Euchromatic histone lysine methyltransferase 2
HDAC1	Histone deacetylase 1
IL-8	Interleukin 8
IL-10	Interleukin 10
LARP7	La-related protein 7
MAD2B	Mitotic spindle assembly checkpoint protein MAD2B
MAN1	Inner nuclear membrane protein Man1
MDM2	MDM2 proto-oncogene
MED15	Mediator complex subunit 15
NEDD4L	Neural precursor cell expressed developmentally downregulated gene 4-like
NEDD8	Neural precursor cell expressed developmentally downregulated protein 8
NF-κB	Nuclear factor kappa-light-chain-enhancer of activated B cells
NOX	NAPDH oxidase
p300	E1A binding protein p300
p32/C1qBP	Complement C1q binding protein
P53	Tumor protein 53
PCGF5	Polycomb group RING finger 5
PKN1 and PKN2	Protein kinase N1 and Protein kinase N2
PLEKHN1	Pleckstrin homology domain containing N1
RSK1	Ribosomal protein S6 kinase, 90 kD, polypeptide 1
RYBP	Ring1 and YY1-binding protein
SATB1	Special AT-rich sequence binding protein 1
SKP1	S-Phase kinase associated TF protein 1
STAT1	Signal transducer and activator of transcription 1
SUPT5H	SPT5 homolog, DSIF elongation factor subunit
TNFα	Tumor necrosis factor α
U2AF35/U2AF1	U2 small nuclear RNA auxiliary factor 1 (35 kDa subunit)

**Table 2:** List of nucleomodulins.

Bacterial Species	Name	Effect	References
*Anaplasma phagocytophilum*	AnkA	Binds to chromatin at AT-rich DNA sequences and nuclear matrix attachment regions (MARs); recruits HDAC1 at the *CYBB* gene promoter and represses *CYBB* expression	[32,33,34,35]
*Bacillus thailandensis*	BtSET	Histone lysine methyltransferase targeting rDNA genes	[59]
*Chlamydia psittaci*	SinC	Interacts with nuclear inner membrane proteins	[39]
*Chlamydia trachomatis*	NUE	Histone lysine methyltransferase targeting host histones	[56,57]
*Coxiella burnetii*	AnkG	Binds to p32; inhibits apoptosis	[106]
*Ehrlichia chaffeensis*	Ank200	Binds to *Alu*-Sx DNA motifs; deregulates expression of several host genes	[42]
	TRP32	Binds to G-rich DNA motifs and chromatin-associated proteins; deregulates expression of several host genes	[46,47,111]
	TRP47	Binds to G + C-rich DNA motifs and chromatin-associated proteins; deregulates expression of several host genes	[48]
	TRP120	Binds to G + C-rich DNA motifs and chromatin-associated proteins; ubiquitin ligase targeting PCGF5 for proteasome degradation; activates several host genes, such as *HOX* genes	[44,45,50,51,52,53]
*Escherichia coli*	Cif	Deamidase targeting the ubiquitin-like protein NEDD8; abolishes the activity of CLR ubiquitin ligases on cell cycle regulators; induces cell cycle arrest	[91,92,93,94]
	EspF	Disrupts a subset of nucleolar factors, such as nucleolin	[109]
	NleC	Protease degrading histone acetyltransferase p300	[97]
	NleG5-1	Ubiquitin ligase targeting MED15 for proteasome degradation	[87]
*Legionella pneumophila*	AnkH	Interferes with RNA Pol II-mediated transcriptional elongation by interacting with LARP7	[102]
	AnkX	Interacts with PLEKHN1	[104]
	RomA/LegAS4	Histone lysine methyltransferase targeting H3K14 and repressing several immune defense genes; in addition, LegAS4 was shown to target rDNA genes in the nucleolus	[58,59,60]
	SnlP	Interferes with RNA Pol II-mediated transcriptional elongation by inhibiting SUPT5H	[103]
*Listeria monocytogenes*	LntA	Inhibits BAHD1 and recruitment of HDACs at interferon-stimulated genes (ISGs), thus activating interferon responses in epithelial cells	[8,22]
	OrfX	Interacts with and promotes RYBP degradation; dampens production of superoxide and nitric oxide in infected macrophages	[27]
*Mycobacterium tuberculosis*	Rv1988	Histone methyltransferase targeting H3R42; represses genes involved in oxidative stress, such as *NOX1, NOX4*	[61]
	Rv3423	Histone acetyltransferase targeting H3K9 or H3K14	[62]
	Rv2966c	DNA methyltransferase targeting cytosines in a non-CpG context	[63]
*Mycoplasma hyorhinis*	Mhy1, Mhy2	DNA methyltransferases targeting cytosines in a CG context	[64]
	Mhy3	DNA methyltransferase targeting cytosines in a GATC context	[64]
*Neisseria meningitidis*	App, MspA	Serine endopeptidases cleaving histone H3; induce increase in dendritic cell death via caspase-dependent apoptosis	[100]
*Orientia tsutsugamushi*	Ank proteins 1U5, 1A, 1B, 1E, 1F, 1U4, 1U9	Contain an F-box domain that interacts with CULLIN-1 and SKP1 of a multiprotein E3 ubiquitin ligase complex; Ank1U5 promotes EF1α ubiquitination and degradation	[89]
	Ank1, Ank6	Promote nuclear export of p65 and inhibits transcription of NF-κB-dependent genes	[101]
*Salmonellaenterica* serovar Typhimurium	GtgA, GogA, PipA	Proteases degrading subunits of NF-kB (p65, RelB et cRel); dampen the inflammatory response	[98,99]
	SspH1	Ubiquitin ligase targeting PKN1 kinase	[71,78,79]
*Shigella flexneri*	IpaB	Pleiotropic functions; delays mitotic progression through APC/Ccdh1 activation and degradation of APC/Ccdh1 substrates	[96]
	IpaH9.8	Ubiquitin ligase targeting splicing factor U2AF35 for proteasome degradation	[71,72,73,74]
	OspB	Binds to Rb transcription factor	[68]
	OspF	Phosphothreonine lyase targeting mitogen-activated protein kinase (MAPK) and inhibiting MAPK-dependent phosphorylation of H3S10; downregulates innate immune response genes; binds to Rb	[65,66,67]
*Streptococcus pyogenes*	SP-STP	Phosphatase; proposed to alter transcription of apoptotic genes	[107]
*Yersinia pestis*	YopM	Targets serine/threonine kinases RSK1 and PKN2; increases transcription of immunosuppressive cytokine genes, such as *IL-10*	[80,81,82,83,84,85,86]

The shaded parts are to better separate the different bacterial species.
